# Vacuum-stent: A combination of endoscopic vacuum therapy and an intraluminal stent for treatment of esophageal transmural defects

**DOI:** 10.3389/fsurg.2023.1145984

**Published:** 2023-02-22

**Authors:** Lisanne M. D. Pattynama, Wietse J. Eshuis, Mark I. van Berge Henegouwen, Jacques J. G. H. M. Bergman, Roos E. Pouw

**Affiliations:** ^1^ Department of Surgery, Amsterdam UMC Location University of Amsterdam, Amsterdam, Netherlands; ^2^ Department of Gastroenterology and Hepatology, Amsterdam UMC Location University of Amsterdam, Amsterdam, Netherlands; ^3^ Cancer Treatment and Quality of Life, Cancer Center Amsterdam, Amsterdam, Netherlands; ^4^ Amsterdam Gastroenterology Endocrinology Metabolism, Amsterdam, Netherlands; ^5^ Department of Gastroenterology and Hepatology, Amsterdam UMC Location Vrije Universiteit Amsterdam, Amsterdam, Netherlands

**Keywords:** VACStent, endoscopic vacuum therapy, esophagus, perforation, anastomotic leakage

## Abstract

**Introduction:**

Endoscopic vacuum therapy (EVT) has gained a greater role in management of transmural defects in the upper gastrointestinal (GI) tract, including anastomotic leakage and esophageal perforation (e.g. Boerhaave syndrome and iatrogenic causes). The vacuum-stent is a new treatment modality, combining the benefits of EVT and an intraluminal stent.

**Patients and methods:**

This prospective case series describes the first ten cases of a transmural defect in the upper GI tract treated with a vacuum-stent in a tertiary referral center. All patients signed informed consent for prospective registration of relevant data on treatment and outcomes in a specially designed database. Outcome parameters were successful closure of the defect, number of endoscopies, duration of treatment and adverse events.

**Results:**

In total, ten patients treated with a vacuum-stent were included. Eight patients had anastomotic leakage after esophageal resection, of whom six were treated with vacuum-sponge and vacuum-stent, and two with vacuum-stent only. One patient had Boerhaave syndrome, treated with vacuum-sponge and vacuum-stent, and one had an iatrogenic perforation during pneumodilation for achalasia, treated with vacuum-stent only. Success rate was 100%, requiring a median of 5 (IQR 3–12) EVT-related endoscopies with a treatment course of median 18 (IQR 12–59) days. One patient developed an esophageal stricture, but no other vacuum-stent related adverse events were observed.

**Conclusion:**

The vacuum-stent, which combines benefits of EVT and an intraluminal stent, shows great feasibility and efficacy in treatment of transmural defects in the upper GI tract. Future studies should point out whether this device can prevent major (re-)surgery in these patients.

## 1. Introduction

Transmural defects in the upper gastrointestinal (GI) tract include anastomotic leakage after upper GI surgery and esophageal perforations e.g., due to iatrogenic causes or Boerhaave syndrome. These defects are associated with severe morbidity and mortality (1, 2). Management of these defects is not standardized and includes conservative, endoscopic and surgical options (3, 4). In recent years, endoscopic treatment has gained a greater role in the treatment of transmural defects in the upper GI tract (5). Endoscopic vacuum therapy (EVT) is a recent development in this field, with very promising outcomes (6–9). EVT can be applied self-fabricated using a suction tube with sponge-material around it, or with a prefabricated sponge (e.g., EsoSPONGE™; Braun B. Melsungen, Germany). The sponge is endoscopically placed over the defect or into the adjacent cavity, and continuous negative pressure can then be applied at the site of the defect. The effect is based on negative pressure wound therapy, resulting in improved wound healing, exudate control and stimulation of perfusion (10). In current literature, success rates are 70 to 100%, with mortality rates of 7 to 18% and complication rates of 10 to 14%, mainly sponge dislocation and stenosis (8, 11).

Recently, a vacuum-stent (VACStent™, MICRO-TECH Europe GmbH, Düsseldorf, Germany) was introduced as a novel device to apply EVT. The VACStent consists of a nitinol covered stent, with a polyurethane sponge attached to its outer surface, and a suction catheter. The stent is placed intraluminally over the defect and connected to a vacuum pump, creating a closed area with negative pressure at the site of the defect. This treatment combines the advantages of negative pressure wound therapy and sealing of the defect by the stent. Furthermore, the negative pressure prevents dislocation of the stent, a common complication when using conventional covered stents, and the stent keeps the esophageal lumen open, allowing for oral intake.

The aim of this study was to describe the first experiences with the VACStent for treatment of transmural defects in the upper GI tract in a tertiary referral center, followed by a discussion and review of available literature on the VACStent.

## 2. Methods

### 2.1. Patients

The first VACStent in the Netherlands was placed in March 2022 at our center. In this case series, all patients treated with VACStent from March 2022 until September 2022, for a transmural defect in the upper GI tract at Amsterdam University Medical Centers were included. A transmural defect was defined as a total disruption of the GI wall, including anastomotic leaks and esophageal perforations, due to Boerhaave syndrome or by iatrogenic causes. Patients were deemed eligible for VACStent treatment by discretion of the endoscopist, and all consecutive patients eligible for VACStent treatment were included. All patients signed informed consent for prospective collection of data regarding EVT treatment in a dedicated database (ClinicalTrials.gov NCT05606822).

### 2.2. Outcome parameters

The primary outcome parameter was successful treatment, defined as closure of the defect with the VACStent alone or in combination with other treatment modalities. Defect closure was confirmed by endoscopy and/or CT imaging. If the defect persisted or increased despite adequate therapy, EVT was considered unsuccessful.

Secondary outcome parameters included use of additional treatment modalities, adverse events within 30 days after VACStent removal and combined in-hospital and 30-day mortality. Adverse events were defined as any events causing deviation of the post-procedural course and were classified by degree of consequences into Grade I to Grade V, using the AGREE classification (12). Furthermore, time from diagnosis to treatment, number of EVT-related endoscopies and duration of EVT were assessed.

### 2.3. Procedures

All endoscopic procedures were performed by interventional endoscopists, using a diagnostic gastroscope. Procedures were performed with the patient under deep sedation or general anesthesia at the discretion of the anesthetist or ICU-physician. During the endoscopy, the defect was inspected thoroughly and cleaned carefully, after which eligibility for VACStent treatment was determined. Contra-indications for VACStent-treatment were: (1) defects larger than 5 cm in length, as the sponge-part of the stent is 5 cm; (2) defects within 2 cm of the upper esophageal sphincter; (3) presence of a contaminated extraluminal cavity requiring extraluminal EsoSponge placement, at discretion of the endoscopist.

**Figure 1:**
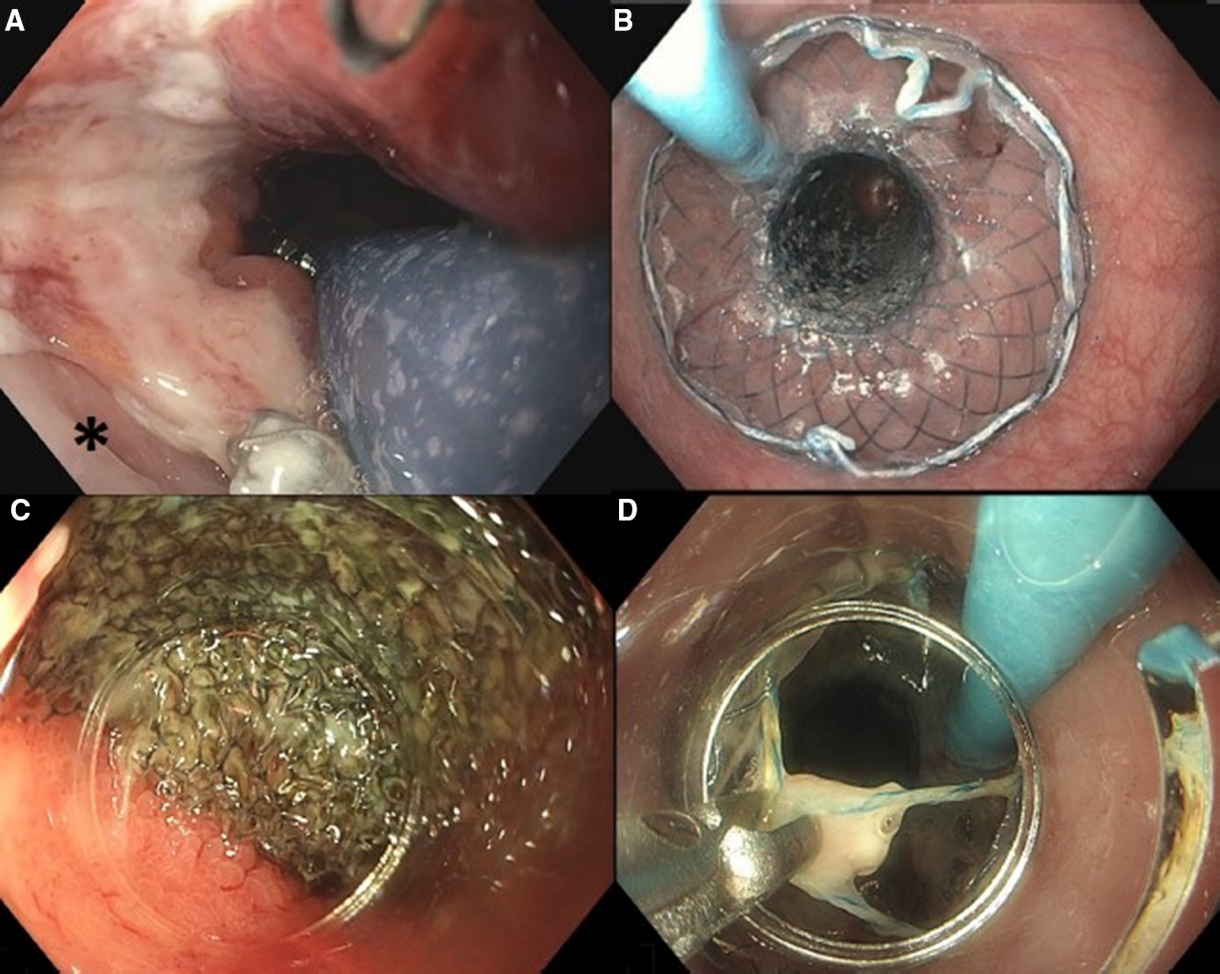
Placement of VACStent: (**A**) positioning of the sponge part of the VACStent over the defect (*). (**B**) Deployed VACStent. Removal of VACStent: (**C**) Separation of VACStent from mucosa using a tapered hood distal attachment cap. (**D**) VACStent removal using grasping forceps.

If VACStent placement was considered feasible, first a stiff guidewire (Ø 0.035”) was placed into the duodenum and the endoscope was removed from the patient. Second, the VACStent introduction device was advanced over the guidewire and introduced into the esophagus. Subsequently, the endoscope was introduced alongside the VACStent introduction device to allow adequate positioning of the VACStent under endoscopic visualization, i.e., with the sponge part of the stent covering the defect (Figure 1A). When the VACStent was in the right position, it was deployed under endoscopic view, *via* the distal release system. During deployment, the position of the VACStent was adjusted accordingly. After the VACStent was completely deployed (Figure 1B), the guidewire and introduction system were removed. Lastly, the blue suction catheter was guided through the nose and connected to a vacuum pump at −125 mmHg.

After one day, negative pressure was decreased to −75 mmHg. On the day of VACStent placement, patients had nil per mouth policy. The day after placement a liquid diet was initiated, which could be expanded to a soft diet if tolerated by the patient. While the VACStent was *in situ*, placement of a feeding tube through the stent was performed if indicated. The VACStent was flushed with 20cc H_2_O 3 times per day to keep the suction catheter open and to prevent ingrowth of the stent.

After 5–7 days, the VACStent was removed (Figures 1C–D). For removal, a tapered hood distal attachment cap (DH-28GR Hood; FUJIFILM Corporation, Tokyo, Japan) was placed on the tip of the endoscope to maneuver the endoscope between the stent and the mucosa. By moving the endoscope from the mucosa to the stent in a downward motion, on all sides, the stent and sponge were loosened from the mucosa. Subsequently, by pulling the string at the proximal site of the stent with a grasping forceps, the VACStent could be safely removed.

After removal, the defect site was inspected to assess closure and to decide if additional EVT was indicated. A new VACStent was placed if necessary.

### 2.4. Statistical analysis

Statistical analyses were performed using SPSS Statistics Version 28 (SPSS Inc., Chicago, IL, USA). Due to the small sample size, descriptive data were expressed as numbers with median and interquartile range (IQR), in case of skewed and even distribution.

## 3. Results

A total of ten patients were treated with EVT using a VACStent (Table 1).

**Table 1:** Patient characteristics and outcomes.

	Total (*n* = 10)	Sponge and VACStent (*n* = 7)	VACStent (*n* = 3)
Age, *median (IQR)*	65 (62–73)	65 (63–74)	65 (56–72)
Male gender, *n (%)*	10 (100)	7 (100)	2 (67)
Etiology of perforation, *n (%)*	10	7	3
Anastomotic leakage	8 (80)	6 (86)	2 (67)
Boerhaave syndrome	1 (10)	1 (14)	0 (0)
Pneumodilation	1 (10)	0 (0)	1 (33)
Success rate, *n* (*%)*	10 (100)	7 (100)	3 (100)
Number of EVT-related endoscopies, *median (IQR)*	5 (3–12)	11 (4–13)	3 (2–4)
Treatment duration in days, *median (IQR)*	18 (12–59)	47 (13–72)	12 (7–14)
Mortality, *n (%)*	0 (0)	0 (0)	0 (0)
VACStent related adverse events, *n (%)*	0 (0)	0 (0)	0 (0)

### 3.1. Anastomotic leakage treated with EsoSponge and VACStent

Five patients, all male with a median age of 65 (IQR 62–75) years, were treated with VACStent for anastomotic leakage, after initial EVT using the EsoSponge. This was in the transition phase, when the VACStent was introduced and was not yet standard initial treatment. All patients underwent an esophagectomy for esophageal cancer, after neoadjuvant chemoradiation. Three patients had a cervical anastomosis and two patients had an intra-thoracic anastomosis. In all cases, suspicion on anastomotic leakage was raised by clinical deterioration and confirmed by CT-scan and endoscopy showing a defect, after a median of 7 (IQR 5–17) days post-surgery.

In all cases, a defect was observed at the site of the anastomosis and in three cases an extraluminal cavity was seen, for which it was decided to place an extraluminal sponge in the cavity. In the other two cases, an intraluminal sponge was placed over the defect. To inspect the defect and exchange the sponge, an endoscopy was performed every 3–4 days in case of an extraluminal sponge, and weekly for intraluminal sponges. In the patients with an extraluminal sponge, the cavity was clean and had become smaller with sponge treatment. In these patients, after 3, 10 and 24 sponge cycles, it was decided to place a VACStent over de remaining defect. In all three cases, the defect was completely closed when the VACStent was removed after one week. In the two patients who initially received an intraluminal sponge, the sponge was removed after one week and a VACStent was placed over the remaining defect. In both cases, the defect had completely healed after one week with VACStent treatment.

In these five patients, median duration of EVT was 21 (IQR 12–77) days.

One patient, a 73-year old man, was treated with a VACStent for a suspected leak at the intra-thoracic anastomosis, 8 days after thoraco-laparoscopic esophagectomy with gastric conduit reconstruction. After 3 weeks of treatment with a total of 3 VACStent cycles, the defect at the anastomosis appeared closed. However, another defect at the distal end of the longitudinal staple line, with an extraluminal cavity was observed, which explained the persisting high infectious parameters and delirium. Due to the location, it was not possible to place a VACStent and it was decided to place an intracavitary sponge. A total of six endoscopies for sponge exchanges were required to heal this second defect.

### 3.2. Anastomotic leakage treated with VACStent only

**Figure 2:**
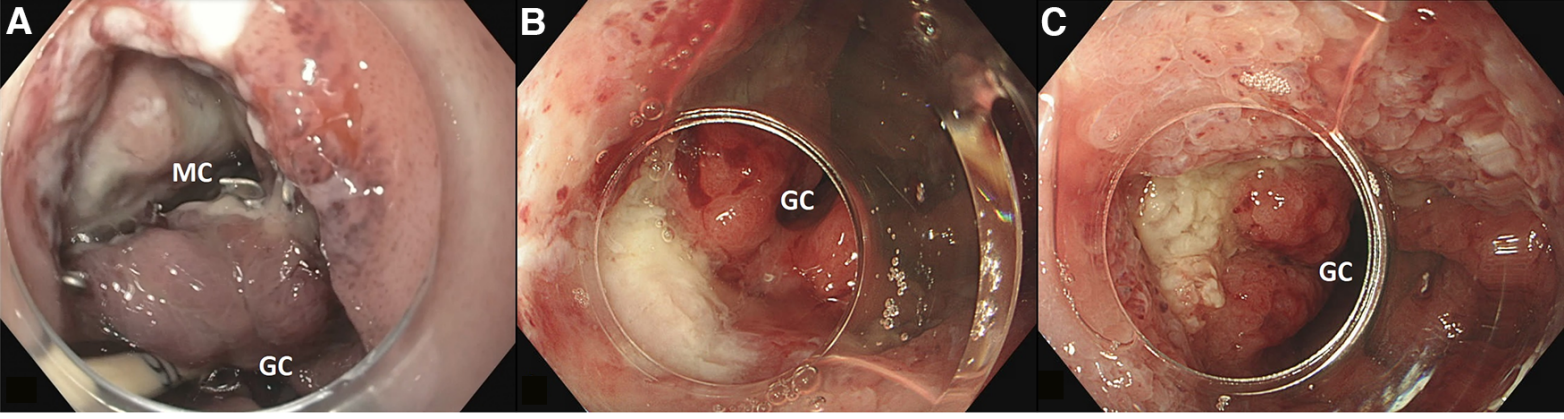
(**A**) large defect with mediastinal cavity (MC) due to anastomotic leakage after esophagectomy with gastric conduit (GC) reconstruction. (**B**) After first VACStent. (**C**) After second VACStent.

Two patients, both male, 72 and 65 years old, developed an anastomotic leak at the intra-thoracic anastomosis after esophagectomy. At 13 and 11 days after surgery, these patients underwent an endoscopy for a suspected anastomotic leak, which was identified in both cases. Subsequently, a VACStent was placed in both patients. In one patient, a large mediastinal cavity was observed (Figure 2A), which could be entered with the endoscope. However, since the cavity was clean, there was no need for extraluminal sponge placement. In this patient, the defect appeared to be closed after one week when the VACStent was removed (Figure 2B). Given the large initial defect it was decided to stay on the safe side and place another VACStent. When this was removed after again one week, the defect had completely healed (Figure 2C).

In the other patient, the defect was closed when the VACStent was removed after one week.

### 3.3. Boerhaave syndrome treated with EsoSponge and VACStent

A 63-year-old male was admitted due to acute chest pain after vomiting. CT-scan confirmed the suspicion of Boerhaave syndrome and endoscopy showed a defect of 4 cm in length in the distal esophagus, with a large and contaminated mediastinal cavity. Due to a sharp angle upwards into the mediastinal cavity, placement of an intracavitary EsoSponge was unsuccessful. Therefore, an intraluminal EsoSponge was placed. After 37 days of treatment, due to stagnant healing tendency observed *via* endoscopy and CT-scan and high infectious parameters, it was decided to perform surgical decortication and placement of an intracavitary muscle flap. Subsequently, intraluminal EVT was continued using sponges. After 23 days, stagnant improvement was observed again and it was decided to place a VACStent, which then had become available. After 14 days of treatment with two VACStents, the defect appeared endoscopically closed, confirmed *via* CT-scan with oral contrast. Three days after VACStent removal and after 2.5 months of hospitalization, the patient was discharged. At 3 months follow-up, the patient had normal intake and there were no signs of stenosis. This case has been published as a video-case report elsewhere (13).

### 3.4. Iatrogenic perforation treated with VACStent only

A 56-year-old female underwent endoscopic pneumodilation for achalasia. After dilation, a transmural perforation of 1 cm was observed in the distal esophagus and a VACStent was placed during the same endoscopy. After 13 days of treatment with two VACStents, closure of the defect was confirmed endoscopically and oral intake was extended successfully.

In this patient, the VACStent bridged the gastro-esophageal junction, resulting in gastro-esophageal reflux that caused nausea and esophagitis that could be treated with proton-pump inhibitors.

### 3.5. Mortality and adverse events

No patients deceased in-hospital or within 30 days after treatment. No adverse events related directly to treatment with VACStent were observed in any of the cases. In one patient with anastomotic leakage, unexpected enlargement of the defect was observed during treatment with sponges, leading to a prolonged treatment course with a total of 24 sponge cycles and one VACStent cycle. Furthermore, one patient with an anastomotic leak at the site of his cervical anastomosis treated with EsoSponge and VACStent, developed an anastomotic stricture, which was treated by multiple endoscopic dilatations and incision therapy.

## 4. Discussion

This paper reports the first ten patients treated with VACStent in the Netherlands at a tertiary referral center. The VACStent is an innovation in EVT for transmural defects in the upper GI tract, as it combines the benefits of negative pressure wound therapy and an intraluminal stent.

The reported cases demonstrate applicability and efficiency of the VACStent in patients with anastomotic leakage and esophageal perforations, including Boerhaave syndrome. Placement of the VACStent was relatively simple, as it uses a commonly known distal release system. Removal of the VACStent was facilitated by using a tapered hood distal attachment cap, that provided the possibility to maneuver the endoscope between the stent and the esophageal wall to safely separate the VACStent from the mucosa.

Furthermore, in these cases, no adverse events or mortality directly related to EVT occurred. One patient developed a refractory stricture. However, this patient had a cervical anastomosis and anastomotic leakage as known risk factors for stricture. As the incidence of anastomotic strictures in cervical anastomosis can be up to 40%, the influence of EVT on the development of this stricture remains unknown (14).

In all but one patient, the defect healed using the VACStent, alone or in combination with sponge therapy and (additional) surgery was avoided. In one patient, with Boerhaave syndrome and no possibility of intracavitary sponge placement, an additional operation for decortication of the lung and filling of the mediastinal cavity with a muscle flap was performed. Most importantly, continuity of the GI tract was preserved in all patients.

Because treatment for transmural defects in the upper GI tract is not yet standardized, the interpretation of the best treatment differs per center and even per physician. Although EVT has proven to be an effective and promising treatment option, intraluminal stenting remains the most commonly used treatment option (15). As there is no conclusive evidence for superiority of these two endoscopic treatments yet, current literature focusses on comparison of the benefits and disadvantages of these treatment modalities (9, 15). The VACStent provides a combination of an intraluminal covered stent and EVT, possibly avoiding the disadvantages of both treatment modalities, while maintaining the benefits. First, due to the negative pressure, the VACStent provides the advantages of EVT, such as exudate control and stimulation of wound healing. Furthermore, the continuous negative pressure applied *via* the suction tube prevents stent dislocation. Moreover, the VACStent provides the possibility of oral intake. Apart from the nutritional benefits, we feel that the possibility of oral intake, even when limited to liquids, caused great satisfaction for the patients. If, for any reason, oral intake is not possible, e.g., when the patient is intubated, a naso-gastric feeding tube can be placed through the VACStent either endoscopically, or blindly. Compared to the EsoSponge treatment, this is an advantage in the absence of a functioning feeding jejunostomy, since it can be quite complicated to place a feeding tube in a patient with an EsoSponge. Furthermore, the presence of a feeding tube next to an intraluminal EsoSponge may prohibit adequate application of the vacuum therapy.

The success rate of the cases described in this paper seems higher than other studies. Since there is extensive expertise in EVT in our center, possibly this expertise (e.g., selection of eligible patients, adequate intervention in case of stagnant healing or complications, efficient logistic arrangements) was a contributing factor to the success rate in this cohort. However, as the VACStent is a relatively new treatment modality, current literature on the subject is scarce and consists of only four small case studies (16–19). First, Chon et al. described a patient with anastomotic leakage successfully treated with VACStent after unsuccessful treatment with over-the-scope-clips (16). Second, de Lange et al. retrospectively reported the successful closure of three defects (anastomotic leakage, Boerhaave syndrome and iatrogenic cause) with VACStent alone or in combination with a covered intraluminal stent (17). Third, Chon et al. retrospectively evaluated 7 cases of anastomotic leakage and esophageal perforations. Of 5 patients treated with only the VACStent, successful treatment was achieved in 4 (80%) (19). Lastly, Chon et al. prospectively described a feasibility study of 20 patients with transmural defects in the upper GI tract, including anastomotic leakages and esophageal perforations, treated with VACStent. Technical success rate was 100% and clinical successful first-line treatment with only VACStent was achieved in 71%. When including cases in which VACStent was used as second-line treatment, success rate was 60%. In 88% of the failed cases, successful treatment was still achieved with additional EVT using sponges (18).

As current literature is scarce, further research should include a larger prospective case series of the VACStent for different indications, such as anastomotic leakage, iatrogenic perforations and Boerhaave syndrome. Then, the best indications and techniques for the VACStent may be assessed, as well as factors contributing to successful treatment with VACStent. For example, in this paper, a combination of VACStent after initial intracavitary EVT with the EsoSponge was used. In case of a large and contaminated cavity, a stepwise combination of these treatment modalities could be beneficial.

Cost-effectiveness analyses are also important to take into account when discussing treatment options for transmural defects in the upper GI tract. Although the initial costs of one VACStent are higher than the cost of one EsoSponge, the VACStent could possibly reduce treatment duration, hospital stay and amount of EVT-related endoscopies. The VACStent has the potential to provide more effective application of vacuum than a sponge, as it establishes an enclosed section with negative pressure around the complete circumference of the esophagus. Furthermore, the nutritional status and quality of life of patients may possibly be better in patients with the VACStent, compared to patients with only sponge therapy. These factors could aid in reducing treatment duration and hospital stay, possibly leading to better cost-effectiveness.

This paper describes the initial experience of a tertiary referral center with the VACStent in the first 10 patients in the Netherlands treated with this new treatment modality for EVT, demonstrating remarkable results. Based on these experiences, the VACStent is a promising treatment option for transmural defects in the upper GI tract without extensive re-operations with additional morbidity.
